# Quantitative Analysis of Lipid Droplet Fusion: Inefficient Steady State Fusion but Rapid Stimulation by Chemical Fusogens

**DOI:** 10.1371/journal.pone.0015030

**Published:** 2010-12-23

**Authors:** Samantha Murphy, Sally Martin, Robert G. Parton

**Affiliations:** The University of Queensland, Institute for Molecular Bioscience and Centre for Microscopy and Microanalysis, Brisbane, Australia; University of Geneva, Switzerland

## Abstract

Lipid droplets (LDs) are dynamic cytoplasmic organelles containing neutral lipids and bounded by a phospholipid monolayer. Previous studies have suggested that LDs can undergo constitutive homotypic fusion, a process linked to the inhibitory effects of fatty acids on glucose transporter trafficking. Using strict quantitative criteria for LD fusion together with refined light microscopic methods and real-time analysis, we now show that LDs in diverse cell types show low constitutive fusogenic activity under normal growth conditions. To investigate the possible modulation of LD fusion, we screened for agents that can trigger fusion. A number of pharmacological agents caused homotypic fusion of lipid droplets in a variety of cell types. This provided a novel cell system to study rapid regulated fusion between homotypic phospholipid monolayers. LD fusion involved an initial step in which the two adjacent membranes became continuous (<10 s), followed by the slower merging (100 s) of the neutral lipid cores to produce a single spherical LD. These fusion events were accompanied by changes to the LD surface organization. Measurements of LDs undergoing homotypic fusion showed that fused LDs maintained their initial volume, with a corresponding decrease in surface area suggesting rapid removal of membrane from the fused LD. This study provides estimates for the level of constitutive LD fusion in cells and questions the role of LD fusion *in vivo*. In addition, it highlights the extent of LD restructuring which occurs when homotypic LD fusion is triggered in a variety of cell types.

## Introduction

Cells store excess fatty acids as neutral lipids such as triglycerides (TG) and cholesteryl esters in unique structures called lipid droplets (also termed lipid bodies). Lipid droplets (LDs) are proposed to form from the cytoplasmic leaflet of the endoplasmic reticulum, and eventually bud to generate discrete organelles bounded by a phospholipid monolayer and containing the cellular machinery required to regulate the deposition and catabolism of neutral lipids [1], [2], [3]. LDs have recently been ascribed many of the functional characteristics of *bona fide* cellular organelles including microtubule-based motility [4], [5], [6], [7],[8], fusion [9], [10], [11] and interaction with other organelles including the ER [12], peroxisomes [13], endosomes [14], mitochondria [15] and caveolae [5], [16].

One of the characteristic features of LDs is their specific size and distribution, which can vary considerably between different cell types. LDs can enlarge through both addition of neutral lipids to pre-existing LDs [17], [18] or through microtubule-dependent fusion [9], [10]. However, when fatty acid levels are elevated LDs frequently form as clusters of similarly sized organelles [5], [19], and in model cell lines such as 3T3-L1 adipocytes large numbers of LDs pack closely together [20], suggesting that unregulated fusion of LDs does not readily occur. The size of LDs relates to both the amount of stored neutral lipid, which is the net result of TG synthesis (lipogenesis) and hydrolysis (lipolysis), and the regulation of lipolysis by external factors. Recent studies imply that LD size and distribution can change in states of lipid related disease. Troyer syndrome, a neurological disease, is associated with a truncated form of the ubiquitin ligase binding protein Spartin (also called SPG20). Overexpression of Spartin induced LD clustering in the perinuclear region whereas knockdown of Spartin increased both the number and size of oleic acid induced LDs [21]. Other types of motor neuron disorders, as well as Berardinelli-Seip congenital lipodystrophy, arise from mutant forms of Seipin (Fld1p), a protein which when deleted in yeast caused fusion of LDs and increased levels of neutral lipids [22]. The neutral lipid storage disease Chanarin-Dorfman syndrome (CDS), which is characterised by the accumulation of LDs in many tissues, is linked to mutations in comparative gene identification-58 (CGI-58). CGI-58 interacts with adipose triglyceride lipase (ATGL), the rate-limiting enzyme for TAG catabolism, and increases its lipase activity [23]. Knockdown of CGI-58 in preadipocytes significantly increased the number of LDs [24] whereas knock-down of ATGL caused a marked increase in average LD size [25], [26]. Similarly, highly enlarged LDs are present in drosophila nurse cell clones with a mutant form of Widerborst, a negative regulator of PI3K/PTEN/Akt associated with diabetes, obesity and some cancers [27]. Together these studies imply constant and tightly controlled regulation of LD size under normal conditions across many cell types, which may break down in disease states.

In view of the number of published studies linking LD size to different functional states, we have investigated the properties of LDs in fibroblasts and in adipocytes by quantitative real-time microscopy. Using the same conditions used in previous studies but refined microscopic techniques we now show that LDs in diverse cell types exhibit low fusogenic activity under normal growth conditions. By screening a variety of pharmacological agents for their ability to alter LD homeostasis we have identified a number of reagents with similar chemical structures that stimulate homotypic fusion of LDs, possibly by localised membrane disruption. This allowed us to validate our refined light microscopic methods to study LD fusion and to carry out a detailed study of the fusion process in living cells. These studies provide new insights into the dynamic regulation of this crucial lipid storage organelle while providing new methods for their study in different pathophysiological states.

## Materials and Methods

### Cell culture

3T3-L1 fibroblasts (American Type Culture Collection, Rockville, MD) were maintained in Dulbecco's modified Eagle's medium supplemented with 10% (v/v) fetal calf serum (Hyclone/ Invitrogen) and 2 mM L-glutamine (Invitrogen), differentiated using insulin, dexamethasone, biotin, and isobutyl-methylxanthine as described previously [28] and used between days 6–12 post-differentiation. Where required, adipocytes were detached from the dishes using 0.05% trypsin and electroporated at 960 µF, 0.16 kV (BioRad Gene Pulser II and Capacitance Extender Plus) for ∼20 ms with 100 µg of DNA on day 8 post-differentiation. NIH-3T3 and BHK-21 cells (American Type Culture Collection, Rockville, MD) were maintained in Dulbecco's modified Eagle's medium supplemented with 10% (v/v) fetal calf serum and 2 mM L-glutamine. Transfection of NIH-3T3s was performed using Lipofectamine Plus (Gibco/Invitrogen) according to the manufacturer's instructions.

### Antibodies, plasmids and reagents

Rabbit anti-Phospho PKA Substrate (RRXS/T) (catalog no. 9624) was obtained from Cell Signaling Technology, rabbit anti-perilipin A (catalog no. P1998) and mouse anti-α-tubulin clone DM 1A (catalog no. T9026) were obtained from Sigma. Alexa488- and Alexa594-conjugated secondary antibodies were obtained from Molecular Probes Inc. (Eugene, OR). Perilipin A-YFP was kindly provided by Dr J. Granneman, Wayne State University School of Medicine, Michigan, U.S.A. Bodipy 493/503 and Nile Red were obtained from Molecular Probes and prepared as saturated solutions in ethanol (working dilution, 1∶200) and acetone (working dilution, 1∶2000), respectively. Oleic acid was obtained from Calbiochem and conjugated to fatty-acid free bovine serum albumin prior to use. Forskolin and ML-7 were obtained from Merck and reconstituted according to manufacturer's instructions. TrypLExpress was obtained from Invitrogen. All other reagents were obtained from Sigma unless stated otherwise.

### Indirect immunofluorescence microscopy and Real-time video microscopy

Indirect immunofluorescence microscopy was performed as described previously [4]. The data were processed using the LSM 510 Meta software (Zeiss), and images were assembled using Photoshop CS3 (Adobe Systems, Mountain View, CA). Cells for real-time microscopy were plated onto glass bottomed tissue culture dishes (MatTek Corp.) or 25 mm round glass coverslips and transferred into CO_2_-independent medium (Invitrogen) supplemented with 0.1% fatty-acid free BSA (Calbiochem). NIH-3T3 cells were incubated for 2 h or overnight in 50 µg/ml oleic acid prior to imaging. When used Bodipy 493/503 was diluted 1∶4000 directly to the imaging medium 10 min prior to commencement of imaging. Reagents were diluted in 1 ml medium and added to 3 ml medium covering cells prior to imaging at a final concentration of 50 µM. Cells were used for real-time data collection for a maximum of 1 hr.

For 4D imaging, time series were collected at 37°C using Axiovert 200 M SP LSM 510 META or 710 META confocal laser scanning inverted microscopes equipped with a 63x oil immersion objective (numerical aperture, NA = 1.4) and a heated stage which held the glass bottomed tissue culture dishes (MatTek Corp.) containing the cells. Z-stack confocal images were taken at 30 s intervals using AIM v3.2 or Zen 2009 software (Zeiss). 4D image analysis of LD motility and clustering in MEF and NIH-3T3 fibroblasts was performed on 30–200 LDs from at least 4 cells from two experiments. 4D image analysis of LD fusion in NIH-3T3s was performed on 50–230 LDs from 5 or more cells across at least 3 replicates using Imaris v7 software to track individual LDs over time. LDs were tracked using the ‘surface’ tool in Imaris with smooth area detail level of 0.1–0.5 µm, size of largest sphere was 0.5–1.5 µm (dependent on average LD size, ∼0.7 µm for NIH-3T3), background subtraction thresholding (local contrast) of 20–40 µm (∼20 µm for MEFs, ∼30 µm for NIH-3T3), default number of voxels, and tracking was performed using a Brownian motion algorithm with a maximum track distance of 20 µm and a maximum gap size of 2 µm. Analysis of 4D images to determine LD surface area and volume is described in detail below (Statistical and mathematical analysis).

2D time series images were collected at 37°C using Personal Deltavision software with an Olympus IX81 inverted microscope fitted with a Roper Cool Snap HQ^2^ monochrome camera, Axyos technologies heated block which held 22 mm round glass coverslips on which the cells were grown and 100x (NA = 1.4) or 60x (NA = 1.42) oil immersion objectives. Image analysis of LD number following treatment with reagents in NIH-3T3s was performed on 10 or more cells over 4 experiments and LD size was measured for the largest LD per cell from 15 cells each from 5 experiments. At least 3 live-cell imaging experiments were performed using each fusogenic reagent. Analysis of LD fusion events triggered by increasing concentrations of H-89 was performed on 3 or more cells for each concentration in two cell types. Alternatively, cells were imaged using an Olympus IX71 inverted microscope fitted with Imago Super VGA 12bit CCD SensiCam (T.I.L.L Photonics), Axyos technologies heated block which held 22 mm round glass coverslips on which the cells were grown and 100x oil immersion objective (NA = 1.4). Time series images were collected using a 470 nm excitation laser filter with an exposure time of 80–100 ms or by bright field microscopy. Image analysis of one and two stage fusion events was performed on 70 or more fusion events from more than 20 cells. Where relevant deconvolution was performed using Personal Deltavision software with the default autoregression algorithm over 10 iterations. All images were converted to 8-bit TIFF files and further analyzed using ImageJ software (National Institutes of Health, Bethesda, MD) or Imaris v7 software. QuickTime videos were assembled using ImageJ 1.37p or Imaris v7, and still images were compiled using Adobe Photoshop CS3.

### Mathematical and Statistical Analysis

Analysis of the volume and surface area associated with LD fusion was conducted on fusion events which were defined either as two droplets in one frame becoming one droplet in the subsequent frame in all dimensions (rendered 3D or ortho slice of the LDs at their largest apparent diameter) or where the surfaces of the two initial droplets became continuous in the resulting droplet (i.e. using perilipin A-YFP). In both cases, the resulting droplet had to remain a single entity in every following frame. As analysis of confocal z-stack images demonstrated that the lipid droplets were spherical, surface area (S.A. = 2πr^2^) and volume (V = 4/3πr^3^) were calculated from the radii of individual droplets, as measured in the x–y plane bisecting the largest apparent diameter of the LD. Subsequently volumes and surface areas of the two initial droplets were compared by linear regression to those of the final droplet. To analyze lipid droplet fusion under various treatments, live cells were imaged and a student's t-test (paired) was used to compare average lipid droplet number per cell between the first and last frames (4–5 videos/treatment).

In fixed cells, measurement of the lipid droplet radii through one x–y plane was used to derive lipid droplet dimensions from micrographs. Between 500–1200 lipid droplets were analysed in >200 cells from randomly chosen fields and differences compared using an unpaired student t-test (two tailed, unequal variance). Analysis of LD number was performed using a particle analysis tool in Image J to identify LDs with a circularity of 0.8–1.

### Western blotting

SDS-PAGE and Western blot analysis was carried out essentially as described previously [4]. Briefly cells were lysed in 10 mM Tris/150 mM NaCl/5 mM EDTA, pH 7.4, containing phosphatase and proteinase inhibitors (Roche), and solubilised in Laemmli sample buffer containing 25 mM DTT. Immunolabeled proteins were visualized using HRP-conjugated secondary antibodies and developed using the Supersignal ECL reagent (Pierce/Quantum Scientific).

## Results

### Analysis of constitutive LD fusion: a quantitative 3D characterization

**Figure 1:**
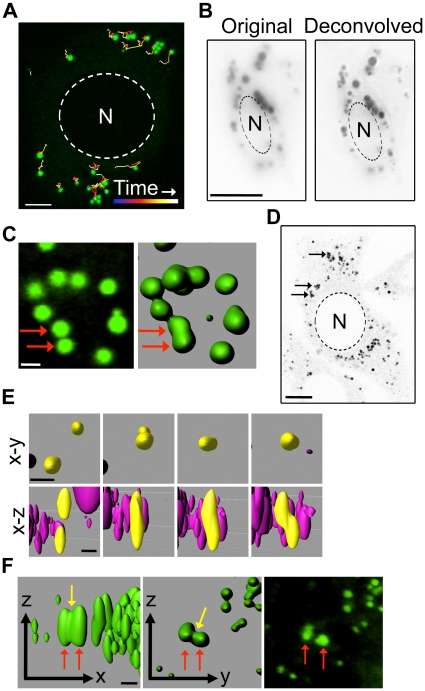
Imaging lipid droplet fusion in NIH-3T3 cells. (a) Bodipy493/503 stained MEFs were imaged using time-lapse fluorescent z-stack confocal microscopy for 30 min. Tracking of the LDs using Imaris software demonstrated that the LDs underwent little directional motility. Tracks are shown through time from blue to white. Bar = 5 µm. (b) Images of Bodipy493/503 stained NIH-3T3s were deconvolved to increase the resolution of individual LDs. Bar = 10 µm. (c) 3D rendering of Bodipy493/503 stained LDs in close proximity can produce a single entity (arrows). Bar = 1 µm. (d) 50% of the LDs in NIH-3T3s are found in clusters (arrows). Bar = 10 µm. (e) Examples of juxtaposed LDs (yellow) which appear to fuse and have a spherical profile as viewed in the x–y plane of rendered LDs but have a highly irregular profile in the x–z plane. Bar = 1.5 µm. (f) The appearance of a ‘waist-like’ structure between two rendered LDs in the x–z and x–y planes is absent when viewed in a single unrendered x–y plane. Bar = 1 µm. N = nuclei.

Despite many reports of LD clustering and studies of LD dynamics in a variety of cell types, few observations of LD fusion have been reported [9], [10], [11], [18], [29], [30]. To examine LD dynamics and interactions we first utilised mouse embryonic fibroblasts (MEFs), which are characterized by extremely thin cytoplasmic regions and so are conducive to live cell imaging. The cells were incubated with 50 µg/ml oleic acid for 2 hr prior to imaging to increase the size and number of LDs, and treated with Bodipy 493/503 to label the neutral lipid components throughout the core of the droplet. Z-stack images of live cells were acquired every 30 s over a 30 min period using time-lapse confocal fluorescence microscopy. Previous studies have shown that LDs undergo microtubule-based motility [4], [5], [6], [31] although the degree of LD movement varies with cell type (for review see [32]). Tracking of individual LDs in MEFs showed an average track length of 9.5 µm±3.3 µm but an average displacement of only 2.2 µm±0.3 µm (Fig. 1a, Video S1), indicating that LDs in MEFs exhibit very little directional movement. LDs were highly clustered with an average of 65% of LDs in close proximity (within 300 nm) to another LD. LDs are commonly visualised by staining the neutral lipid core with fluorescent dye that allows clusters of LDs to be readily resolved into individual components when imaged through the x–y axis. The resolution of individual LDs could be further enhanced by deconvolution (Fig. 1b). However, the resolution of individual LDs within a cluster was lost when z-stack images were collapsed (in the z axis) or surface rendered. These techniques generally resulted in clusters of LDs appearing as large irregular structures and LDs in close proximity to one another to appearing as a single entity (Fig. 1c). In order to analyse LD dynamics in Bodipy-stained cells, a combination of 2D and 3D visualisation was used. Despite the extensive LD clustering observed in MEFs no LDs appeared to undergo fusion in the observed time frame.

Studies by Bostrom and colleagues reported constitutive LD fusion in NIH-3T3 fibroblasts [10]. To determine if the difference in reported LD fusion in NIH-3T3s and the lack of observed LD fusion in MEFs could be related to a difference in LD motility or clustering, analysis of LD dynamics in NIH-3T3s was performed as described above (using conditions identical to those reported by Bostrom et al [10]). LDs in NIH-3T3 fibroblasts were found to travel an average of 6.6±0.4 µm with an average displacement of 2.9±0.4 µm, similar to LD motility observed in MEFs. NIH-3T3 cells also showed a similar level of LD clustering as seen in MEFs with an average of 52% of LDs in close proximity to one another (Fig. 1d). To examine the dynamics of LD fusion, z-stack images of live cells were acquired over 5 min periods using time-lapse confocal fluorescence microscopy and subsequently collapsed (in the z plane) or surface rendered to produce 2D and 3D images, respectively. Analysis of LD interactions was performed using the criteria for fusion as outlined by Bostrom et al [10] (two initial droplets are not more than 3.5 µm apart, the volume of the resulting droplet must not exceed the combined volume of the two initial droplets by more than 50% and must be present in the timepoint following fusion without a change in volume). Many of the LDs were closely associated with other LDs during the capture time and 2.1±0.9% of the total number of LDs appeared to fuse as defined by the above criteria. However, analysis in the z-plane of the apparently fused LDs revealed irregular profiles, not readily discernible in the x–y plane, which were not consistent with the more spherical profile of unfused droplets (Fig. 1e, left panel) or droplets formed when fusion was triggered (see below for examples of triggered fusion). Further analysis of each ‘fused’ droplet observed in the 3D rendered cells showed that 69.9% of ‘fused’ droplets were clearly separate entities when viewed in unrendered x–y planes through a z-stack. In addition, although we were able to observe a ‘waist-like’ structure between LDs prior to fusion in 3D rendered cells, these structures were not apparent when the LDs were analysed in single x–y planes (Fig. 1f). In light of the discrepancies between ‘fused’ droplets when visualised in different planes, and in comparison to unequivocal fusion events observed when the cells were treated with a variety of agents (see below) we established the following criteria to define LD fusion in this system; [1] fused LDs persist as a single entity for the remainder of the capture time (which must be no less than half the image capture time, here 2.5 min), as expected for a stable fusion event, [2] over that period no discontinuity between fused LDs is observed in either the x–y or the x-z-axis and [3] the resulting droplet becomes increasingly spherical as fusion progresses (irregularities in LD shape do not persist for more than 2 min). Using these criteria, in our hands no unequivocal fusion events were observed in NIH-3T3 cells imaged over 5–30 min, suggesting that constitutive homotypic LD fusion is a rare event and is likely to be tightly regulated.

### Screening for agents with lipid droplet fusion activity

To begin to examine the dynamic regulation of LDs we screened a number of inhibitors of protein kinases (eg. protein kinase A, PKA; extracellular signal-regulated kinase, ERK) or upstream receptors (β-adrenergic receptors, β-ARs) for their effects on LD size and number in NIH-3T3 fibroblasts (Table 1). To screen for LD fusion, NIH-3T3s were incubated in 50 µg/ml oleic acid overnight to generate large numbers of mature LDs, treated with reagents for 1 hr, fixed and stained with Bodipy493/503. Randomly selected micrographs were deconvolved and the cells analysed for LD number and size.

**Table 1:** Agents screened for lipid droplet fusion activity.

Reagent	Targets
H-89	**PKA**, S6K1, ROCK-II, MSK-1, PKBα, AMPK, PKG, CaCMK-II, MLCK, ERK1/2, PRK2, RSK1/2, PKD1
ML-7	**MLCK**, MSK-1
Propranolol	B-adrenergic receptors
SR 59230A	B3-adrenergic receptor
U0126	**MEK**, PRAK, SAPK2a/2b
KT5720	**PKA**, PDK-1, MEK, MSK-1, PKBα
BIM-I	PKC isoforms

Primary targets highlighted in bold. PKA, cAMP-dependent protein kinase; S6K1, p70 ribosomal protein S6 kinase 1; ROCK-II, Rho-dependent protein kinase II; MSK-1, mitogen- and stress-activated protein kinase 1; PKBα, protein kinase B α; AMPK, AMP-activated protein kinase; PKG, cGMP -dependent protein kinase; CaCMK-II, calcium/calmodulin-dependent protein kinase II; MLCK, myosin light chain kinase; ERK1/2, extracellular-signal-related kinase 1/2; PRK2, protein kinase C-related protein kinase 2; RSK1/2, p90 ribosomal protein S6 kinase; PKD1, serine-threonine protein kinase D1; MEK, MAPK kinase (also called MKK); PRAK, p38-regulated/activated kinase; SAPK2a, stress-activated protein kinase 2a (also called p38); SAPK2b, stress activated protein kinase 2b (also called p38β2); PDK-1, 3-phosphoinositide-dependent protein kinase 1; PKC, protein kinase C; BIM-I, bisindolylmaleimide I.

**Figure 2:**
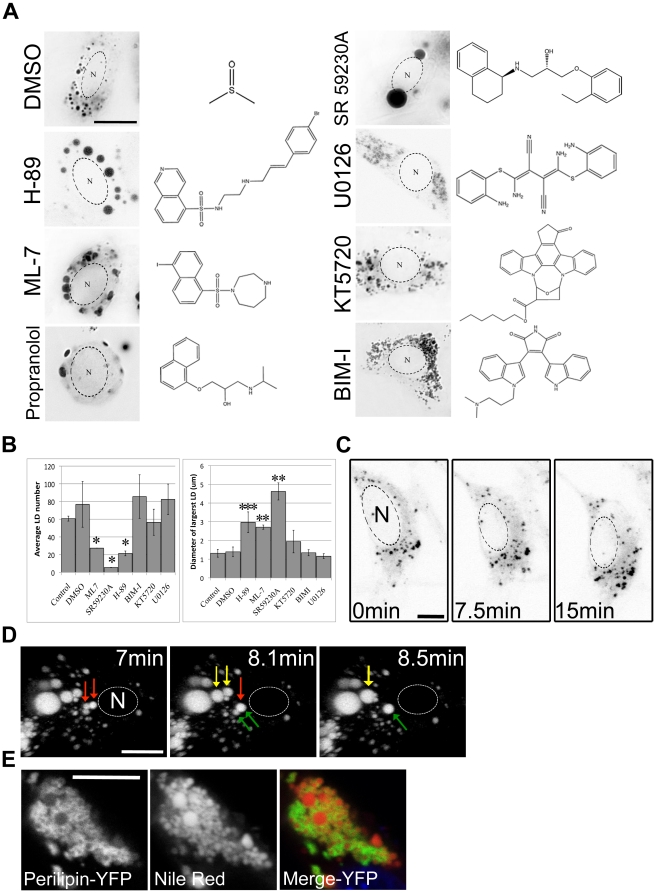
Lipid droplet fusion can be triggered specifically by chemicals. (a) Representative images NIH-3T3 cells treated with a variety of chemical reagents and stained with Bodipy493/503. Bar = 10 µm, N = nucleus. The chemical structure of each reagent appears beside the image. (b) Random micrographs from each reagent treatment were analysed for both LD size and number. Fusogenic reagents caused a decrease in LD number concurrent with an increase in LD radius. Error bars represent the S.E.M of at least 12 cells from 3 or more replicates, *p<0.0005, **p<0.005, ***p<0.05. (c) Prolonged imaging of LDs using Bodipy493/503 in NIH-3T3 cells failed to detect LD fusion. Bar = 5 µm, N = nucleus (d) Time-lapse imaging of NIH-3T3 cells stained with Bodipy493/503 demonstrated that multiple fusion events were triggered by addition of 50 µM H-89. Coloured arrows indicate fusing pairs of LDs. Bar = 10 µm (e) Plin A-YFP was expressed in NIH-3T3 cells and the LDs detected using Nile Red. Bar = 10 µm.

The initial reagent screen revealed that treatment with H-89, ML-7, propranolol and SR 59230A resulted in a significant reduction in LD number with a concurrent increase in average LD size, indicative of LD fusion (Figure 2a, b). To confirm that the LD remodelling seen in the fixed cells was the result of LD fusion, rather than increased lipid accumulation in LDs or inhibition of LD fission or catabolism, time-lapse z-stack imaging of live cells was performed. No detectable alteration in the structure of LDs, including fission or fusion events, was observed in NIH-3T3 cells stained with Bodipy493/503 during prolonged imaging (up to 30 min) under control conditions (Fig. 2c). However, treatment with 50 µM H-89 (Figure 2d, Video S2), 50 µM ML-7, 50 µM SR 59230A and 200 µM propranolol triggered fusion of adjacent LDs as defined by the above criteria, where the LD contents merged to form a single droplet with a spherical profile in all planes, as well as cell rounding (data not shown). Fusion was only observed in cells treated with reagents at well above physiologically relevant concentrations (≥50 µM). Treatment of NIH-3T3 and baby hamster kidney (BHK-21) cells with increasing concentrations of H-89 showed inhibition of phospho-PKA substrate phosphorylation was at least partly achieved with 10 µM H-89 whereas homotypic LD fusion only occurred at concentrations of 50 µM or above (Figure S1a). Furthermore, the rate of LD fusion appeared to be concentration dependent as 100 µM H-89 triggered a 6-fold increase in the number of fusion events over cells treated with 50 µM H-89 (Figure S1b). In addition, LD fusion events were not observed in cells treated with less than 200 µM propranolol (data not shown) despite inhibition of both the β1- and β2-ARs being achieved at much lower concentrations [33]. Together these data suggest that LD fusion was triggered by some other property of the reagents, for example biophysical properties, rather than simply the inhibition of their respective targets. Despite the ambiguity surrounding the mechanism of action of these drugs, we have identified H-89, ML-7, propranolol and SR 59230A as effective triggers of homotypic LD fusion.

To fully validate homotypic fusion of LDs (where the surfaces of the initial droplets become continuous and the contents merge) from possible docking events (where the LDs are in close contact but do not become one droplet), Perilipin A-YFP (Plin A-YFP) was used as a marker of the LD surface. A monolayer marker produces more reliable results than staining the LD core with a fluorescent dye, particularly as imaging through the LD in single x–y planes allows demonstration of the continuation of the membranes during LD fusion. When expressed in NIH-3T3 cells Plin A-YFP localised to the surface of all detectable LDs as judged by Nile Red staining (Fig. 2e) and a net increase in number and mean LD size was observed (data not shown), consistent with previous studies [34]. However, overexpression of Plin A-YFP did not cause LD fusion within the timeframes studied here, as judged by phase contrast microscopy and by the use of the Bodipy dye (data not shown). Simultaneous phase-contrast and fluorescent imaging showed adjacent cells with and without expression of Plin A-YFP underwent LD fusion with similar kinetics when treated with 50 µM H-89 (Data not shown), demonstrating that expression of Plin A-YFP had no observable influence on LD fusion. Further analysis of fusion between Plin A-YFP-labelled LDs showed continuation of the LD membrane following fusion.

### Stimulated fusion of lipid droplets in adipocytes

**Figure 3:**
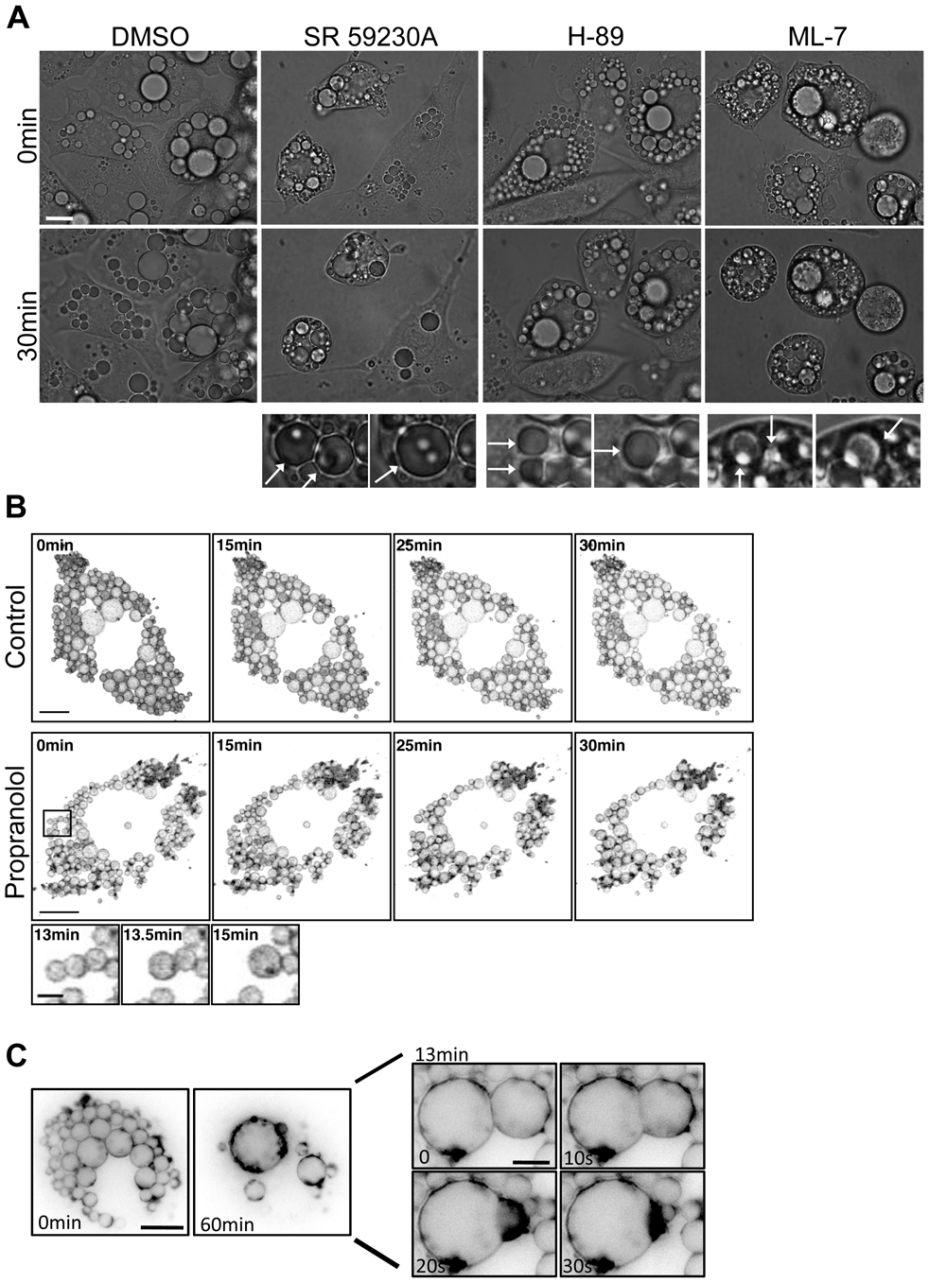
Fusion can be triggered in 3T3-L1 adipocytes. (a) 3T3-L1 adipocytes were replated and imaged in real-time using bright field microscopy for a total of 30 min. No LD fusion events were observed when cells were treated with vehicle (DMSO). However, addition of 50 µM SR 59230A, 50 µM H-89 or 50 µM ML-7 triggered fusion of LDs (arrows in blow-up) and cell rounding. Bar = 20 µm. (b) Perilipin A-YFP was transiently expressed in 3T3-L1 adipocytes and imaged by real-time microscopy in the absence or presence of 200 µM propranolol. Z-stack confocal microscopy images were acquired every 30 s over 45 min and rendered to produce a 3D image of the cell. There was no significant motility or detectable fusion of labelled LDs in control cells. Following treatment with 200 µM propranolol, multiple LD fusion events were observed in over 80% of the cells expressing perilipin A-YFP. Many LDs underwent multiple fusion events, highlighted in the sequential fusion of three LDs (insert). Bar = 10 µm, insert = 2 µm. (c) 3T3-L1 cells transiently expressing Plin A-YFP were imaged in a single plane during treatment with 50 µM SR 59230A. The membranes of the two fusing LDs became continuous within 10 s and the LD cores had merged within 30 s. Bar = 20 µm, blow-up = 10 µm.

We next investigated the characteristics of chemical-induced LD fusion in 3T3-L1 adipocytes. Unlike fibroblasts, which contain tens of small LDs (∼1 µm diameter), 3T3-L1 adipocytes contain a number of large, perinuclear LDs (5–10 µm diameter), which makes them ideal for detailed analysis of the dynamics of monolayer-monolayer fusion, as occurs between fusing LDs. Live cell imaging was performed to determine if the fusogenic reagents identified in the fibroblast screen could trigger LD fusion in 3T3-L1 adipocytes. Analysis of brightfield time-lapse images of 3T3-L1 adipocytes showed low motility and no fusion events under control conditions (Fig. 3a). However, treatment with 50 µM H-89 (Video S3), ML-7 (Fig. 3a) or SR 59230A (Video S4) triggered the rapid fusion of adjacent LDs (Fig. 3a). Further analysis of 3T3-L1 adipocytes treated with H-89 showed that LD fusion was triggered in all cells observed. LD fusion appeared temporally regulated, with fusion occurring 16.6±2.6 min after the addition of H-89 and reaching an apparently stable state (no further fusion events) after 42.4±3.1 min. Detailed analysis was also performed on both single slice and z-stack real-time images of 3T3-L1 adipocytes expressing Plin A-YFP. In control cells Plin A-YFP labelled LDs showed very little motility and no detectable fusion within a 45 min time period (Fig. 3b). In contrast, over the same time period treatment with 200 µM propranolol resulted in multiple LD fusion events in over 80% of cells analysed (Fig. 3b, Video S5, detail Video S6). Fusion triggered by propranolol was temporally regulated, similar to that observed in H-89 treated cells. Individual fusion events occurred rapidly initiating approximately 45 min after addition of propranolol, before the LDs again entered an apparently stable state. Fusion triggered by SR 59230A (50 µM), a selective β3-AR antagonist, also followed a similar pattern with fusion ceasing 34.6±4.1 min after initiation. Imaging of Plin A-YFP labelled LDs in a single x–y slice clearly showed continuation of the membrane during fusion triggered by SR 59230A (Fig. 3c).

In addition to triggering LD fusion, treatment with H-89, ML-7, propranolol and SR 59230A all induced cell rounding. To determine if cell rounding itself, through loss of adhesion to the substratum or disruption of the cytoskeleton, could trigger LD fusion 3T3-L1 adipocytes were treated with nocodazole to disrupt the microtubule network, cytochalasin D to disrupt the actin network or trypsin to detach cells from the substratum. Treatment of 3T3-L1 adipocytes with nocodazole disrupted the microtubule cytoskeleton but did not alter LD morphology (Figure S2a) or increase LD volume (Figure S2b). No LD fusion was observed in time-lapse images of 3T3-L1 adipocytes treated with the above reagents for up to 30 min (data not shown). In addition, the microtubule network was not required for LD fusion to occur. Disruption of the microtubule network with nocodazole did not prevent LD fusion in cells subsequently treated with 200 µM propranolol (Figure S2). Together this data suggests that LD fusion is not a direct result of cell rounding.

Taken together the results suggest that defined chemicals can trigger LD fusion in a specific fashion but that this effect is likely to be unrelated to their properties as kinase inhibitors. This ability of these fusogenic chemicals to trigger LD fusion in 3T3-L1 adipocytes was subsequently used as a model system with which to study the dynamics of LD fusion *in vivo*.

### Lipid droplet fusion occurs in two stages

**Figure 4:**
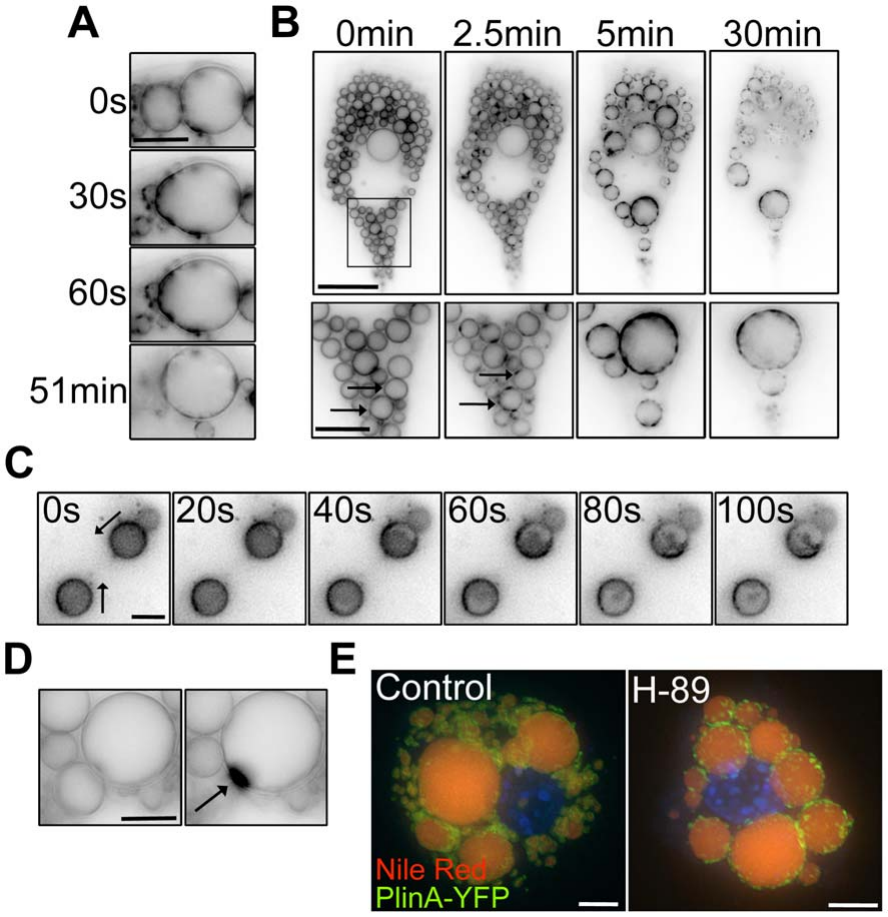
Lipid droplet fusion occurs in two stages and the lipid droplet surface is disrupted upon treatment with fusogenic reagents. (a) 3T3-L1 adipocytes expressing perilipin A-YFP were imaged in real-time for a total of 30 min. Analysis of single fusion events in 3T3-L1 adipocytes treated with 50 µM SR 59230A showed that the initial fusion (defined by the continuity of the LD membranes) was completed within 1 frame (30 s) whereas the reformation of a spherical structure could take several minutes. Bar = 10 µm. (b) H-89 (50 µM final concentration) was added directly to the medium whilst imaging (Bar = 20 µm). Prior to any LD fusion being observed, Plin A-YFP redistributed into dense patches on the LD surface (arrows). Bar = 10 µm. (c) In adipocytes treated with SR 59230A the directional loss of Plin A-YFP across the surface of the LDs was observed. Bar = 10 µm. (d) An example of the appearance of a discrete, intensely fluorescent structure at the site of LD fusion. Bar = 10 µm. (e) 3D rendering of z-stack images show the LD core (stained with Nile Red) remains spherical although the surface has been disrupted (as seen by Plin A-YFP labelling). Bar = 5 µm.

Real-time fluorescence microscopy of live cells was undertaken to examine the characteristics of homotypic LD fusion. 3T3-L1 adipocytes with and without ectopic expression of Plin A-YFP were used as model systems in which to analyse individual fusion events triggered by the addition of H-89 or SR 59230A. Detailed analysis of fusion by fluorescence microscopy in Plin A-YFP expressing cells and by brightfield microscopy in untransfected cells revealed two populations of fusing LDs; those in which fusion was completed and the resulting droplet was spherical within 10 s (1 frame) (72.9% and 75.3% of total fusion events in untransfected and transfected cells, respectively) and those where fusion appeared to occur in two stages: an initial rapid fusion of the surfaces of the two LDs to form a continuous membrane within 10 s (1 frame), followed by a slower merging of the contents and the reformation of a single round LD which occurred over 10 s to 17.5 min (27% and 24.7% of total fusion events in untransfected and transfected cells, respectively) (Fig. 4a, Video S7). In both transfected and untransfected cells, the majority of two-stage fusion events reformed spherical droplets within 100 s of initial fusion of the membranes (90% in untransfected cells, 67% in Plin A-YFP expressing cells), although a small number of droplets in the Plin A-YFP expressing cells took up to 17.5 min to regain a spherical shape, as judged by the circularity of the LD surface in a single x–y plane.

### Lipid droplet surface rearrangement occurs both prior to and following lipid droplet fusion

Under control conditions Plin A-YFP in fibroblasts and adipocytes uniformly localised to the LD surface and formed a continuous ring in a single x–y slice (Fig. 4b). Following treatment with fusogenic reagents a marked redistribution of Plin A-YFP over the surface of the LDs was observed, with the formation of dense patches of Plin A-YFP prior to LD fusion (Fig. 4b, Video S8), or the directional redistribution of Plin A-YFP on LDs that did not subsequently undergo fusion for the duration of imaging (30 min) (Fig. 4c). Post-fusion, Plin A-YFP was observed to redistribute into large, discrete structures on the surface of the fused LD, often appearing fixed at the site of fusion (Fig. 4d). Prolonged imaging of fused droplets showed that these dense Plin A-YFP structures persisted for over 30 min. Despite the redistribution of Plin A-YFP on the LD surface, the LD itself remained intact and spherical, as determined by 3D rendering of fixed cells and visualisation of the lipid core by Nile Red staining (Fig. 4e).

### Volume but not surface area is conserved during lipid droplet fusion

**Figure 5:**
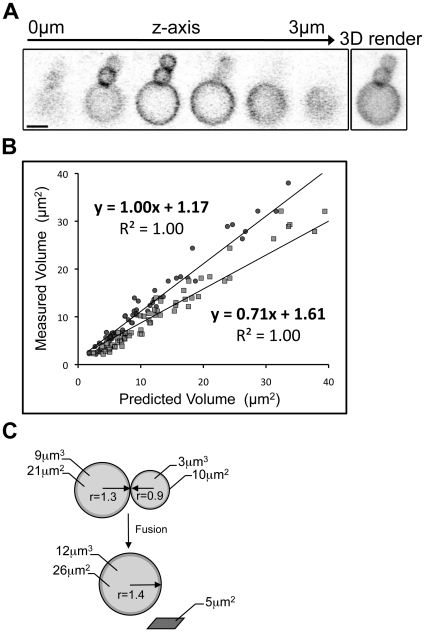
Modelling and analysis of individual lipid droplet fusion events. Sequential confocal images taken through the z-axis and subsequent 3D rendering of the z-stack clearly show the spherical shape adopted by perilipin A-YFP containing LDs in 3T3-L1 adipocytes. Bar = 2 µm. (b) LD volumes were calculated following 60 individual fusion events and plotted against the predicted volume, assuming either conserved volume (dark grey circles) or conserved surface area (light grey squares) of the initial LD. The data were analysed by linear regression and the best fit in each case designated by a solid line. The equation of the trend line, and R-squared value is displayed adjacent to each data set. Analysis clearly demonstrated that the best fit was attained when the volume was conserved (slope of the line = 1). (c) Schematic modelling of LD fusion demonstrates the excess surface area generated by fusion when volume is conserved.

LDs assume a spherical shape in 3T3-L1 adipocytes consistent with a low surface area to volume ratio, as seen in 3-dimensional rendering of z-stack confocal images (Fig. 5a). Fusion of two spheres must result in a sphere with either the same volume or the same surface area as the two initial spheres. To determine whether surface area (membrane) or volume was conserved during LD fusion, we analysed the change in volume and surface area of resulting fused LDs relative to the two donor LDs. We measured the volumes and surface areas of 60 pairs of donor LDs in live cells and used these values to predict the volume of the resulting LD, assuming either volume or surface area conservation. To determine how closely the predicted volumes matched the actual volumes, the two sets of predicted volumes were plotted against the measured volumes of the resulting LDs (Fig. 5b). In the case where the volumes of the two donor LDs were conserved, we showed a linear relationship between the predicted and measured volumes with a slope of 1, demonstrating that the predicted volumes of the resulting LDs closely match the actual volumes. However, where the surface areas of the donor LDs were conserved, the slope of the trend line was 0.71, showing that the predicted volumes of the resulting LDs were larger than the actual volumes. Together, this demonstrated that the volume of the two donor LDs was conserved during fusion (Fig. 5b). Modelling of fusion between LDs of a range of sizes indicated that the fused LDs achieved a minimal volume with a resulting loss of up to 22% of the combined starting surface area, within 30sec of the fusion event (Fig. 5c). As LDs are constrained within a limiting phospholipid monolayer these data suggest that either phospholipids are rapidly removed from the LD following fusion, or that significant compaction of the LD phospholipid monolayer can occur.

## Discussion

The processes governing LD biology are currently under intense scrutiny as many metabolic diseases have been associated with changes in LD distribution, function and size. The formation of LDs in some cell types, especially macrophages, liver and muscle, is associated with the progression of increasingly common metabolic diseases such as insulin resistance, type II diabetes and cardiovascular disease. In order to elucidate the mechanisms behind changes in LD morphology in disease states it is necessary to develop a more complete understanding of the fundamental biology of LDs. Of particular interest is the regulation of LD size. It is well documented that an increase in fatty acids leads to the formation of tightly packed clusters of similarly sized LDs [5], [11], [18], [19] which are able to function as discrete organelles [6]. However, fusion of LDs has been observed very rarely [18], [29], [30], with few exceptions [9], [10], [11]. This suggests that if fusion of mature LDs were to occur, it would be an infrequent and highly regulated event. As LD size (volume) is very consistent within a specific cell type, fusion would have to be balanced by rapid removal of neutral lipids from the newly formed droplet or as yet unobserved subsequent fission events (which would require additional monolayer formation). Although homotypic LD fusion has been observed in 3T3-L1 cells undergoing differentiation [35], this process is likely to be tightly controlled as the large LDs present in mature differentiated 3T3-L1 adipocytes pack tightly together without resulting in constitutive LD fusion. Regulated fusion of LDs has been shown to occur in Drosophila S2 cells lacking Cct1 or Cct2, proteins involved in phospholipid synthesis. Knockdown of other genes involved in phospholipid biosynthesis (CK, HLH106, SCAP and FAS) also resulted in an increase in LD size concurrent with a decrease in LD number, presumably due to LD fusion [11]. From studies in COS7 cells, treatment with fatty acid or palmitate induces LD formation which is associated with an increase in the amount of phosphatidylcholine (PC) (as well as TG and diacylglycerol) in the LD fraction [18]. As PC is limiting in Cct1 knockdown cells, it has been suggested that a decrease in PC, along with an increase in the relative amount of phosphatidylethanolamine may promote LD fusion to decrease the surface area to volume ratio, limiting the requirement for PC for membrane synthesis [11].

Although unregulated LD fusion has not been readily observed in most cell types, recent studies by Bostrom et al [9], [10] show constitutive fusion of approximately 15% of the total LDs in NIH-3T3 cells at any given time. These fusion events have been shown to be microtubule dependent and mediated by SNARE proteins [9], [10]. Although this study showed a striking difference in the apparent size and distribution of the LDs in SNAP23 knockdown cells compared to control conditions [9], our results suggest that this treatment may alter clustering or docking of LDs rather than fusion. The methods described here can now be applied to the question of SNARE-mediated LD regulation and to the study of other proteins proposed to regulate homotypic fusion of LDs.

As no examples of unequivocal LD fusion were observed under control conditions, we screened for reagents that may modulate LD fusion. Here we have shown that a variety of pharmacological reagents at used at high concentrations can trigger homotypic LD fusion in both fibroblasts and adipocytes. Treatment with 50 µM H-89, 50 µM ML-7, 50 µM SR 59230A and 200 µM propranolol each triggered rapid fusion of adjacent LDs. Each of these reagents have different documented primary targets and a number of listed off-target effects [36], [37], [38], [39]. Comparison of the structures of the fusogenic reagents to those which did not trigger fusion revealed the presence of a naphthalene group (two benzene rings joined in the ortho position) in three of the four fusogenic reagents; H-89, ML-7 and propranolol. The presence of a naphthalene group in a molecule creates a highly hydrophobic region which has been shown to insert into the hydrophobic core of a lipid bilayer [40], [41], [42]. A study of the mechanism of action of propranolol on artificial membranes showed that propranolol disrupts the outer phospholipid monolayer of liposome bilayers prior to the formation of ‘worm-like micelles’ [43]. In addition, treatment of liposomes with low concentrations of propranolol resulted in stabilisation of the liposomes, suggested to be the effect of interactions between the propranolol and proteins on the liposome surface. Treatment of the same liposomes with high concentrations of propranolol destabilised the liposomal membranes causing them to lyse [44]. This may explain the observation that fusion occurs in 3T3-L1 adipocytes treated with 200 µM propranolol but not 100 nM propranolol (data not shown). Together this data leads to the possibility that some of the fusogenic reagents found in our screen trigger LD fusion at high concentrations at least in part through insertion into, and local disruption of, the LD phospholipid monolayer. This hypothesis is in agreement with the observation that triggered LD fusion only occurs between adjacent LDs, rather than trafficking of LDs towards one another prior to fusion as would be expected for functionally regulated LD fusion. However, a similar mechanism of local membrane disruption may be utilised by the cell on a small scale to allow homotypic fusion to occur in specialised scenarios, such as the reformation of large LDs from microLDs produced during stimulated lipolysis in adipocytes. Importantly, although we were not able to discern the mechanism of action of the fusogenic reagents, this study demonstrates that LD fusion can occur and we were able to use this system to perform detailed analysis of the unique dynamics arising from fusion between monolayer membranes within the cell.

Using the fusogenic reagents found in our screen allowed us to characterise LD fusion. In the majority of treated cells the LDs underwent rapid homotypic fusion with adjacent LDs, reaching an apparently stable state approximately 30 min after fusion was first observed. This temporal regulation may be the result of saturation of a system or pathway in the fusion process, or the resulting droplets being too far apart to undergo further fusion events. Approximately a quarter of the fusion events recorded progressed via a two-step process where the initial membrane fusion was observed within 10 s, followed by the gradual merging of the LD contents over 10 s to 17.5 min. Addition of fusogenic reagents also induced LD surface rearrangement prior to and following LD fusion, which may be further indication that the fusogenic reagents trigger fusion by disrupting the LD membrane. Plin A-YFP on the LD surface was seen to form dense patches upon the addition of fusogenic reagents, leaving areas of the surface without Plin A-YFP. Furthermore, following fusion Plin A-YFP often decorated the site where fusion had occurred with stable, large and intensely fluorescent patches. One possibility is that these intense patches of Plin A-YFP labelling denote flaps of excess membrane generated through LD fusion. Modelling of LD fusion based on measurements of LD size during fusion showed that the volume of the two initial droplets was conserved in the final fused droplet, which can be predicted to generate excess membrane. Regulated LD fusion in control cells could therefore provide membrane phospholipids if required for other cellular processes. This would also generate larger LDs, which have a more efficient surface area to volume ratio for lipid storage. Although constitutive LD fusion in basal cells will remain controversial, we have shown that LD fusion can occur in both fibroblasts and adipocytes though in our hands this occurs only when triggered by the addition of fusogenic reagents and is likely to be the result of LD surface disruption. However, the possibility remains that under specific conditions cellular processes may disrupt the LD surface in order to facilitate regulated fusion. This raises the possibility that tightly regulated partial fusion with bilayered organelles could also occur [45] We propose that this mechanism could allow precise regulation of the ‘hemifusion’ between LDs and other organelles, as has been suggested to occur between LDs and early endosomes [14], facilitating the direct transfer of LD-associated proteins or lipid components for either oxidation in mitochondria, efflux from the cell surface, conversion to phospholipids in the ER or the transfer of fatty acids from the late endosomes/lysosomes. Spatiotemporal regulation of LD fusion suggests a mechanism by which monolayer-monolayer and monolayer-bilayer interactions could be controlled and represents a unique and powerful model system with which to study membrane interactions *in vivo*.

## Supporting Information

Figure S1 **The fusogenic effect of H-89 is concentration dependent.** (a) BHK cells were treated with increasing concentrations of H-89 and subsequently stimulated with Forskolin and IBMX for 30 min. Whole cell lysates were western blotted for phospho-PKA substrates and tubulin. Representative of 3 experiments. (b) Time-lapse imaging of NIH-3T3s stained with Bodipy493/503 (Bar = 5 µm) showed a 6-fold increase in the number of fusion events per cell in cells treated with 100 µM H-89 over cells treated with 50 µM H-89. Error bars represent the S.E.M of at least 10 cells per condition from two experiments. *p<0.05.
(TIF)

Figure S2 **Cell rounding does not trigger LD fusion.** (a) 3T3-L1 adipocytes were treated for 30 min with 44 µM nocodazole and either fixed directly in ice-cold methanol for 3 min, or further treated with 200 µM propranolol for 1 hr prior to fixation. Cells were labelled for α-tubulin and perilipin A, and the nuclei detected using DAPI. Bar = 20 µm (b) Cells treated as in (a) were fixed in 4% PFA, labelled for perilipin A and stained with DAPI. The average volume of LDs/cell is the percentage change relative to the control volume in two different experiments. Error bars represent the S.D. of 500–1200 cells from at least 3 replicates. *p<0.001.
(TIF)

Video S1 Mouse embryonic fibroblast treated with oleic acid overnight and stained with Bodipy493/503. Z-stack confocal image taken at 1 frame per 30 s, volume rendered. Coloured lines indicate LD movement over time.
(MOV)

Video S2 NIH-3T3 treated with oleic acid overnight and stained with Bodipy493/503. Z-stack confocal image taken at 1 frame per minute, volume rendered. Treatment with 50 µM H-89 triggered fusion of adjacent LDs (examples circled).
(MOV)

Video S3 3T3-L1 adipocytes imaged by brightfield microscopy at 1 frame per 10 s. Treatment with 50 µM H-89 triggered dramatic fusion of adjacent LDs.
(MOV)

Video S4 3T3-L1 adipocytes imaged by brightfield microscopy at 1 frame per 10 s. Treatment with 50 µM SR 59230A triggered extensive fusion of adjacent LDs (examples circled).
(MOV)

Video S5 3T3-L1 adipocyte expressing Plin-YFP imaged at 1 frame per minute. Z-stack confocal image, rendered. Treatment with 200 µM propranolol triggered fusion of adjacent LDs.
(MOV)

Video S6 Detail of LD fusion taken from Video S7 shows sequential fusion of adjacent LDs.
(MOV)

Video S7 Expression of Plin A-YFP in a 3T3-L1 adipocyte showed continuation of the LD membranes during fusion triggered by treatment with 50 µM SR 59230A. Fluorescent image taken at 1 frame per 10 s.
(MOV)

Video S8 Plin A-YFP redistributed on the LD surface in a 3T3-L1 adipocyte both prior to, and following LD fusion triggered by treatment with 50 µM SR 59230A. Fluorescent image taken at 1 frame per 10 s.
(MOV)
