# Supplementary figures and images for: Quantitative Analysis of Lipid Droplet Fusion: Inefficient Steady State Fusion but Rapid Stimulation by Chemical Fusogens
Source: PLoS One. 2010 Dec 23;5(12):e15030. doi: 10.1371/journal.pone.0015030 (PMC3009727; doi:10.1371/journal.pone.0015030)

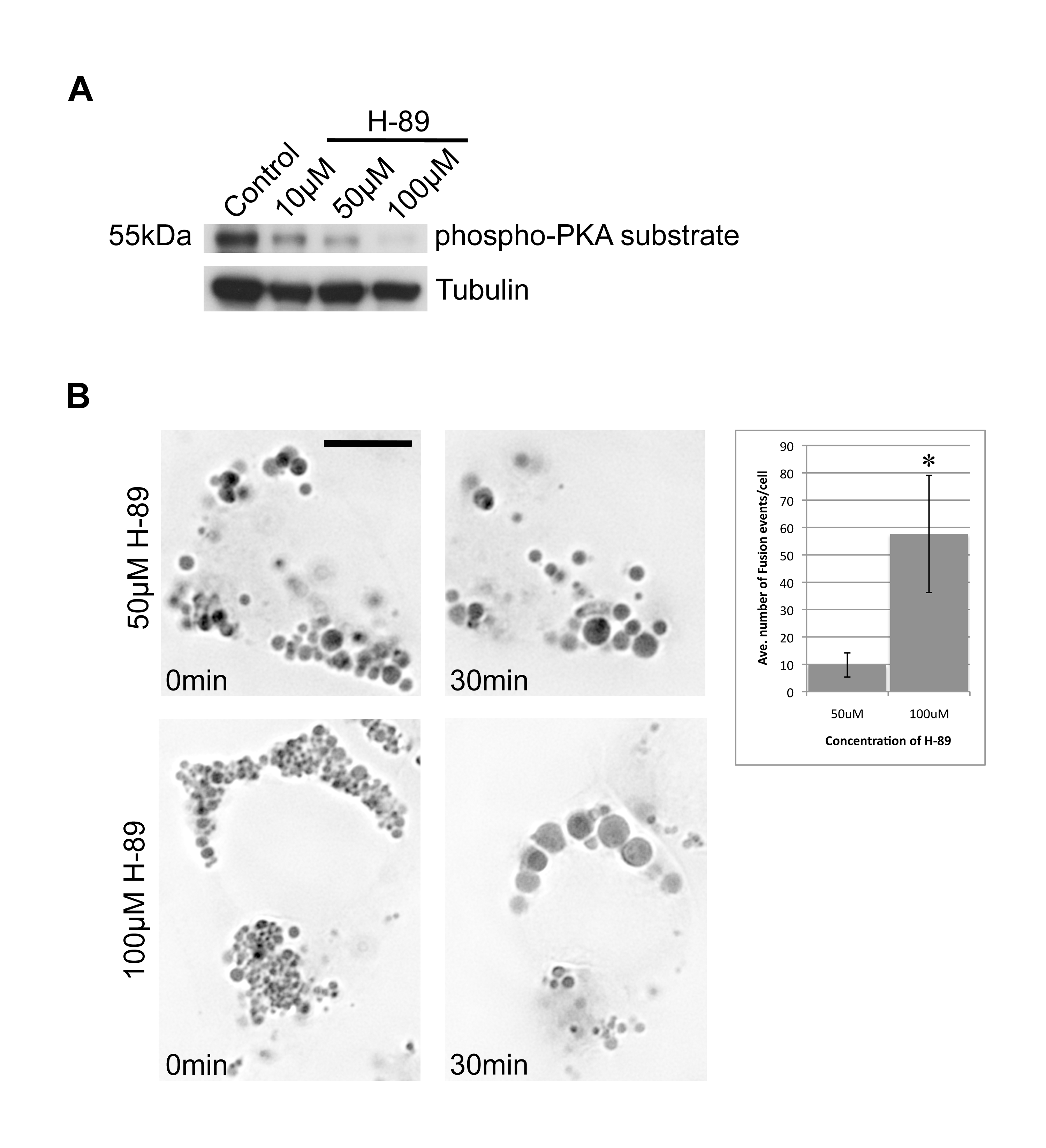

Supplement: Figure S1 — The fusogenic effect of H-89 is concentration dependent. (a) BHK cells were treated with increasing concentrations of H-89 and subsequently stimulated with Forskolin and IBMX for 30 min. Whole cell lysates were western blotted for phospho-PKA substrates and tubulin. Representative of 3 experiments. (b) Time-lapse imaging of NIH-3T3s stained with Bodipy493/503 (Bar = 5 µm) showed a 6-fold increase in the number of fusion events per cell in cells treated with 100 µM H-89 over cells treated with 50 µM H-89. Error bars represent the S.E.M of at least 10 cells per condition from two experiments. *p<0.05. (TIF) [file pone.0015030.s001.tif]

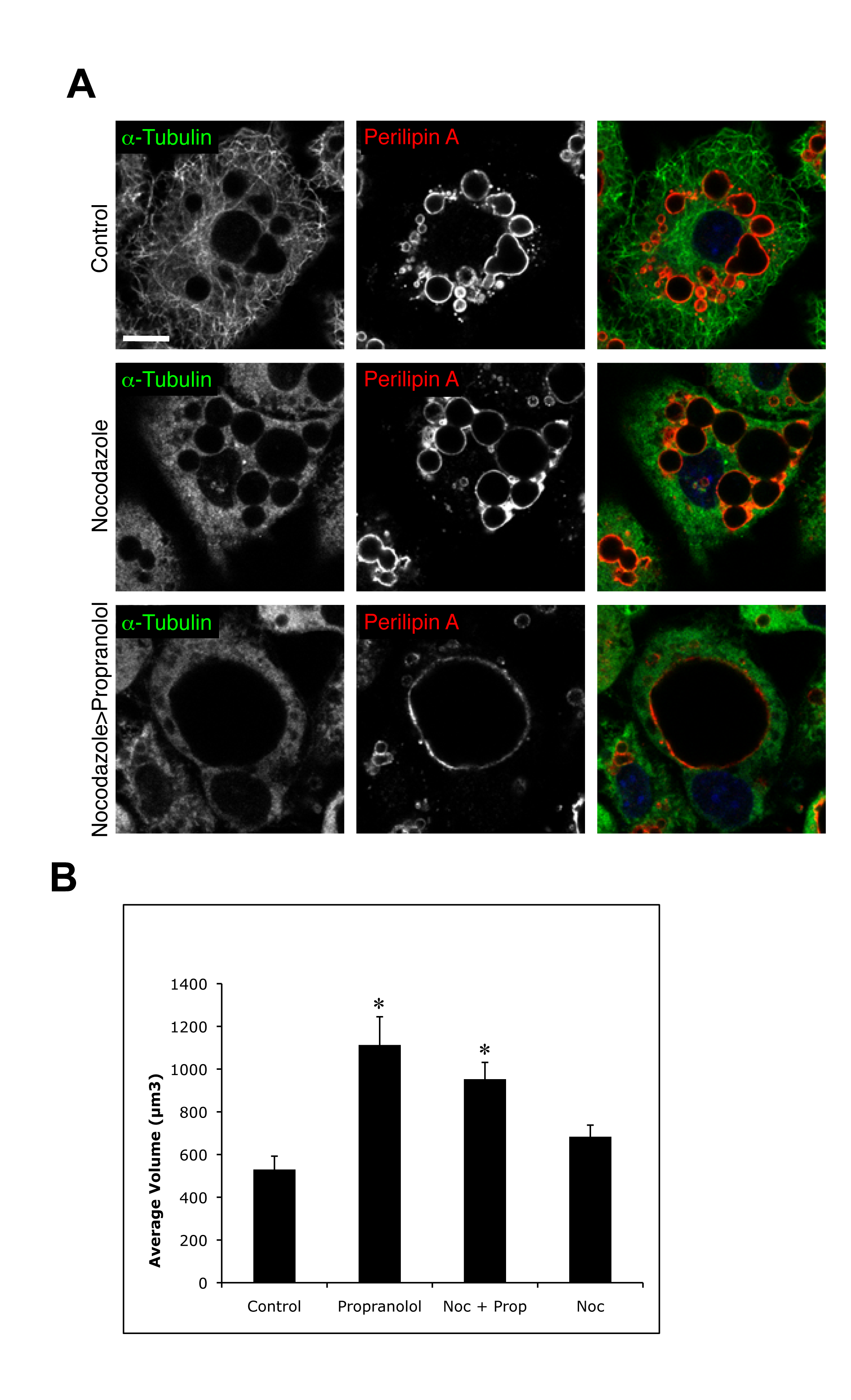

Supplement: Figure S2 — Cell rounding does not trigger LD fusion. (a) 3T3-L1 adipocytes were treated for 30 min with 44 µM nocodazole and either fixed directly in ice-cold methanol for 3 min, or further treated with 200 µM propranolol for 1 hr prior to fixation. Cells were labelled for α-tubulin and perilipin A, and the nuclei detected using DAPI. Bar = 20 µm (b) Cells treated as in (a) were fixed in 4% PFA, labelled for perilipin A and stained with DAPI. The average volume of LDs/cell is the percentage change relative to the control volume in two different experiments. Error bars represent the S.D. of 500–1200 cells from at least 3 replicates. *p<0.001. (TIF) [file pone.0015030.s002.tif]
